# Understanding Smart Home Sensor Data for Ageing in Place Through Everyday Household Routines: A Mixed Method Case Study

**DOI:** 10.2196/mhealth.5773

**Published:** 2017-06-13

**Authors:** Yasmin van Kasteren, Dana Bradford, Qing Zhang, Mohan Karunanithi, Hang Ding

**Affiliations:** ^1^ Adaptive Social and Economic Systems Commonwealth Scientific and Industrial Research Organisation Dutton Park Australia; ^2^ Australian e-Health Reseach Centre Commonwealth Scientific and Industrial Research Organisation Herston Australia

**Keywords:** activities of daily living, aged, remote sensing technology

## Abstract

**Background:**

An ongoing challenge for smart homes research for aging-in-place is how to make sense of the large amounts of data from in-home sensors to facilitate real-time monitoring and develop reliable alerts.

**Objective:**

The objective of our study was to explore the usefulness of a routine-based approach for making sense of smart home data for the elderly.

**Methods:**

Maximum variation sampling was used to select three cases for an in-depth mixed methods exploration of the daily routines of three elderly participants in a smart home trial using 180 days of power use and motion sensor data and longitudinal interview data.

**Results:**

Sensor data accurately matched self-reported routines. By comparing daily movement data with personal routines, it was possible to identify changes in routine that signaled illness, recovery from bereavement, and gradual deterioration of sleep quality and daily movement. Interview and sensor data also identified changes in routine with variations in temperature and daylight hours.

**Conclusions:**

The findings demonstrated that a routine-based approach makes interpreting sensor data easy, intuitive, and transparent. They highlighted the importance of understanding and accounting for individual differences in preferences for routinization and the influence of the cyclical nature of daily routines, social or cultural rhythms, and seasonal changes in temperature and daylight hours when interpreting information based on sensor data. This research has demonstrated the usefulness of a routine-based approach for making sense of smart home data, which has furthered the understanding of the challenges that need to be addressed in order to make real-time monitoring and effective alerts a reality.

## Introduction

An aging population is a challenge to current models of health care delivery and engagement [1]. Not only will there be a greater proportion of older people in the general population, but the prevalence of chronic disease in the aged will increase health care costs and put pressure on health care services. To service the future needs of an aging population, the elderly will increasingly be encouraged to remain in the community or age in place [2,3]. Smart homes can facilitate early intervention and aging in place, particularly for people living alone. In Australia, 1 in 4 people aged 65+ years live alone [4]. In-home sensors can provide continuous monitoring to facilitate early intervention through alerts to carers (family, relatives, health care professionals) of acute medical events (ad hoc alerts) and patterns suggesting cognitive or physical decline (notification alerts).

Real-time alerts and visual presentation of data from smart homes sensors are a work in progress. In a 3-month UK pilot study, Sixsmith [5] tested alerts based on unusual motion and absence of motion. Alerts comprised an automated phone call to the residents, and, if they did not answer a call, to a carer. The system produced 61 alerts, 46 of which were false alerts. Trial participants felt that while a few false alarms were acceptable, too many were viewed as intrusive and ongoing false alerts undermined faith in the system. In a Japanese pilot study of smart homes, Ohta et al [6] set up alerts based on transition time between rooms, which were classified as normal or unusual. Unusual transition movement (eg, staying longer than normal in a room) triggered a phone call or an email to a carer. The number of alerts and false alerts was not reported.

Visual presentation is also important for making smart homes a reality. The amount of data generated by continuous monitoring systems can result in information overload [7] unless summarized in a way that allows carers to understand the situation at a glance and make judgment calls about the required response [8,9]. Visual summarization of sensor data is difficult given the need to present spatio-temporal data from a multitude of sensors [10].

In the Sixsmith pilot study [5], no visual information was available to the participants or carers. Ohta et al [6] proposed an example of a display based on room transitions so carers could visually check the current location of the resident based on a limited recent history (a few hours) of movement between rooms. Kaye et al [11], in an extensive smart home trial in Oregon, illustrated 180 days of daily room movement using spiral plots in a 24-hour format. Although cyclical patterns emerged in the spiral plots for strict daily routines, it was not possible to identify or interpret more fluid routines.

Routines are described as “strategically designed behavioral patterns (conscious and subconscious) used to organize and coordinate activities along the axes of time, duration, social and physical contexts, sequence and order” [12]. Human activity is structured into routines, which reflect the cyclical nature of human biological and social behavior, which is organized around a 24-hour clock [13-16]. Although routines are not inherently good or bad, changes to routines can be significant. Maintenance of routines, especially those associated with the activities of daily living, is essential for independent living [17-19]. Furthermore, as aging involves the inevitable decline in cognitive and physical function leading to modification of daily routines to match the altered functionality [20,21], changes to routine may be indicators of underlying issues such as decreases in cognitive health and well-being [22,23]. This study investigated (1) whether routines can be extracted from sensor data and (2) how routines can advance interpretation of sensor data to provide triggers and thresholds for real time, reliable ad hoc alerts.

## Methods

### Smart Home Sensors

This study presented results from a pilot of Smarter Safer Homes testing ubiquitous home monitoring for the elderly, as described in Zhang et al [24] and Bradford et al [25,26]. Smart homes were installed with a range of sensors. However, this study only looked at data from motion and power sensors. The full list of sensors is shown in Table 1. The study was conducted in accordance with Health and Medical Research Human Research Ethics. The participants in the study agreed to the installation of in-home sensors.

**Table 1:** In-home sensors for residents.

Sensors	Trigger for sensor firing	Place of installation	Sensor data upload	Data type
Passive infrared motion sensors	Motion within 5 m	Wall (near ceiling) in all rooms	Sends ad hoc as status change	Binary
	Current draw of appliances	Wall power outlets	Pushes 1-minute data every 5 minutes	KwH to binary
Circuit meter	Current draw of stove or oven	Switchboard	Pushes 1-minute data every 5 minutes	KwH to binary
Accelerometer	Movement	Under bed	Sends ad hoc as status change	Binary
Reed switches	On breaking of circuit	Exit doors. Kitchen or bedroom doors	Sends ad hoc as status change	Binary
Acoustic sensor	Water flow	Kitchen	Sends ad hoc as status change	Binary
Environmental sensors	Continuous data collection	Kitchen, bathroom, laundry	Pushes 1 reading every minute	Temperature and humidity
Electronic thermometer	Daily recording of temperature^a^	Indoor usage	Sends ad hoc after measurement	Temperature
Glucometer of blood	Daily recording of blood pressure^a^	Indoor usage	Sends ad hoc after measurement	Glucose
Electronic scales^b^	Daily recording of weight^a^	Indoor usage	Sends ad hoc after measurement	Weight
iPad and Web portal	Residents were given an iPad for their personal use and to consult an app on which summary sensor data appeared and diary for health measurements			

^a^Residents would vary in the regularity and consistency of use of these devices.

^b^Information sent directly to database without the need for user data entry.

### Sensor Data and Installation

The data used in this study were collected from passive infrared motion sensors and power use sensors located in participants’ lounge, kitchen, bedroom, and bathroom. These sensors were chosen because they were the most reliable and provided the bulk of the data. Motion sensors send data whenever motion is detected. Motion sensors fire on detection of movement, but cannot distinguish between types of movements. Also, movements have to be sufficiently large for sensors to fire. Power use sensors were connected to all key appliances in the kitchen and lounge. Power use sensors send data (KwH) every 5 minutes.

Sensors were installed and maintained by a local technician. The technician was very friendly, and his visits were appreciated by all residents. The most common complaints involved battery replacement and flickering lights on motion and power sensors. Duct tape was used to hide the light on motion sensors, and residents were shown how to switch the light off on power sensors.

### Data Preparation and Cleaning

Time (GMT) and date stamped data from motion and power sensors were relayed to a Web-based database and downloaded to Microsoft Access. Each entry identified the residence, room, sensor type (motion or power), and description (eg, kettle). Time was converted into local time, which comprised 16 days of AEST (GMT+10) and 175 days AEDT (GMT+11).

Data were analyzed using Microsoft Access. Prior to analysis, data were cleaned. Standby power was removed for appliances such as televisions and microwaves (based on low wattage) and then converted into binary data (in use or not in use). Missing data because of sensor battery failure or absence from home were identified. Absences over 24 hours were noted, but did not negatively affect daily data or cumulative data. Battery failure was distinguishable because of normal movement in other rooms. For some motion sensors, a notable increase in sensor firing prior to battery failure caused high outliers. High outliers could also occur on days residents received guests or visitors. Mean replacement was used for high outliers (±3×IQR).

Continuous streamed data 24/7 for 3 residents over 181 days resulted in 345,470 data entries. Data were presented as 24-hour radar plots, which reflected daily patterns of movement and power based on cumulative frequency of sensor firing. Data were grouped by hour (± 30 minutes). Radar plots reflected the build-up and change in pattern of movement or power over time. Sharp spikes in the outlines indicated strict adherence to time schedules.

### Participants

Residents of an aged care facility living in independent units self-selected for participation in the pilot. To be eligible to participate in the pilot, participants had to be aged over 70 years and have no home care arrangements. Participants with cognitive difficulties were also excluded. Of those who self-selected (N=23), 17 signed consent forms; however, 3 residents withdrew before the sensors were installed. Retention of participants in longitudinal trials with the elderly was problematic for reasons including morbidity, mortality, relocation, or other. Over the course of the 180 days of the trial, there were further withdrawals. We collected 7 complete sets of data (sensor and interview data at 3 time points); however, only 5 residents were eligible for this study. Participants living in dual occupancy were excluded because multiple occupancy was problematic for interpreting data from the motion sensor used in this trial [11]. The 5 eligible participants were aged between 79 and 88 years (Mean 83.6, SD 3.8). There were more female (n *=* 4) than male participants (n *=* 1). Two participants listed primary or secondary school as their highest level of education, 2 had non university certificates or diplomas, and only 1 had a university education. Of these 5, 3 residents were chosen as case studies because they presented opportunities to explore different challenges [27-29]. Pseudonyms were used to ensure anonymity of participants.

### Interview and Personal Data

Three interviews were conducted 2, 6, and 8 months after sensor installation. Interviews were recorded and transcribed. Relatives or friends were present at most interviews and contributed to the discussion. Relatives of case study participants were also interviewed separately.

Participants answered a brief questionnaire on preference for routinization using a shortened version of Reich & Zautra’s scale [30] (Table 2). The shortened scale consisted of 8 context-relevant statements requiring a true or false answer (eg, “I do pretty much the same thing every day”). A percentage preference for routine was calculated based on the number of positive (preferred routine) responses to these statements.

**Table 2:** Preference for routinization and outings based on qualitative data and scores for routinization.

Pseudonym	Score^a^	Description	Activity	Regular outings		
				Family	Care facility	Community
Rupert	n/a^b^	Set daily routines that vary little from day to day	Fairly sedentary or cerebral	Low but regular	High	Low
Elizabeth	100%	Routine varies by day of week	Active or always busy	Med	Med	High
Jacqui	63%	Flexible daily routines that also vary by day of week. Travels a lot.	Very active or restless energy	High (daily)	Low	Low

^a^Shortened scale of 8 items [30].

^b^Only answered true to 1 of the 5 statements.

## Results

### Routines and Routinization Are Highly Individual

Although daily activities comprise many common elements such as eating and sleeping, the way in which activities are organized in time reflects the uniqueness of individual routines [31,32]. The 3 cases reflect very different routines. Rupert, a widower, has an unvarying and sedentary routine. He always has lunch at the residence restaurant (Monday to Saturday), he takes regular daily walks, and on Sundays, he goes to church and has lunch with his son’s family. When at home, he spends most of his time in the lounge, watching informational programs. Elizabeth, a widowed housewife, is very busy but has a regular routine. Elizabeth attends bridge club 3 times a week. She also meets up with her daughter at least once a week. At home, she is very busy cooking and housekeeping. Rupert, who had a very regular routine, only answered 1 of the 8 items. He agreed to the statement “I generally stick to a certain scheduled once I have started it.” Elizabeth scored 8 out of 8 (100%) on the preference for routinization scale [30] (Table 2).

Jacqui, a widow and former secretary, travels a lot and is frequently away from home for as many as 14 days at a time. She is also not often at home during the day, and her routine is irregular. She scored 5 out of 8 items (63%), indicating a low preference for routinization. Her grandson said: “she’s got her routines but sometimes she just doesn't do them.”

### Sensor Data Match Self-Reported Routines

**Figure 1:**
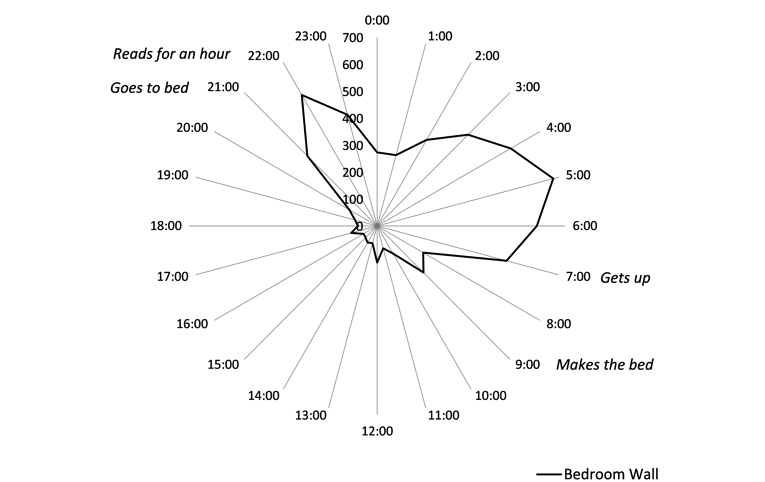
Elizabeth: Radar plot showing cumulative motion in bedroom by time of day (24/7 over 181 days) as indicated by the time of firing of motion sensors. Data is grouped by hour (±30 minutes).

**Figure 2:**
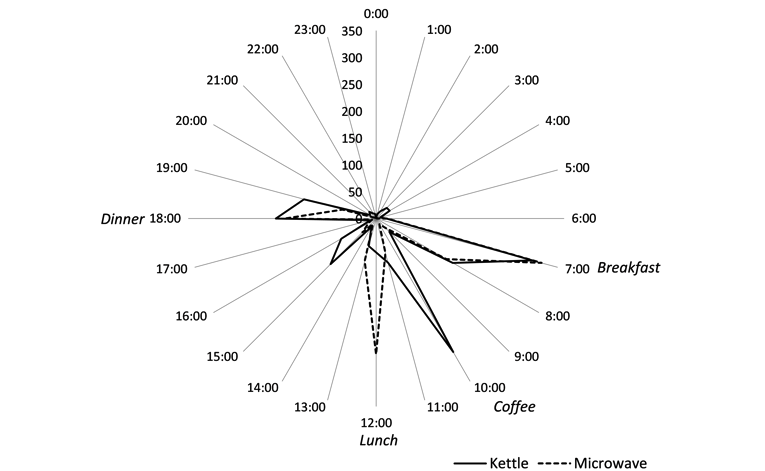
Elizabeth: Radar plot showing kitchen appliance power use (kettle solid line, microwave dotted line) by time of day based on cumulative frequency (24/7 over 181 days). Text in italics reflects the routine activity as per Elizabeth's self-reported routine.

Results comparing routines constructed from sensor data closely matched resident’s self-reported routines, as illustrated in Figure 1 and Figure 2. The congruency between sensor data and self-reported routines was apparent for all 3 residents, suggesting that data were accurate. In addition to matching the self-reported routines, further observations were possible from the radar plots. Elizabeth commented on being a restless sleeper, and this was confirmed by the bedroom motion sensor, especially between 3 am and 6 am and kettle use between 2 pm and 3 pm. Spikes in kettle use around 3:30pm suggested that on the days she was home, she had afternoon tea. However, without the context from self-reported routines, sensor data can be misinterpreted. Elizabeth’s television power use suggested that she spent all day watching television, but her interview revealed that she had the television on all day for company as the voices gave her the feeling that “somebody’s here with me.” Even when watching television, she was generally knitting or crocheting. Self-reported routines personalized the information, giving a much richer insight into Elizabeth’s life.

### Detecting Change in the Activities of Daily Living Routines

Times series data are traditionally analyzed by examining the component parts: cyclical, seasonal, trend, and irregular variations. In these data, cyclical variation shows as recurring weekly patterns by day of week. Seasonal variation in data includes annual recurrent patterns of temperature and timing of sunset and sunrise by time of year. Trend data is longitudinal change over time, which is important for the detection of slow-onset gradual decline in physical or cognitive abilities over and above cyclical and seasonal data. Finally, irregular variation can explain ad hoc disruption to routines such as illness or death of a spouse.

#### Cyclical: Understanding Cultural Patterns

Cyclical variation results from the organization of days of the week into work and rest days as well as the annual cycle of holidays. These cultural rhythms appear in the routines of the elderly as a continuation of past habits or indirectly through their contact with relatives. Jacqui’s routine absences from home are dependent on the schedule of her relatives. The most direct impact of cultural rhythms on the elderly is the scheduling of community events such as church and bridge club.

**Figure 3:**
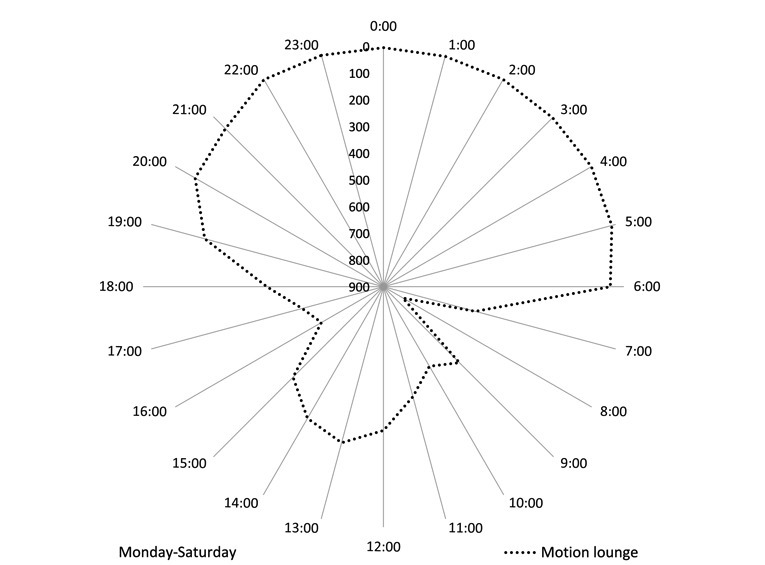
Rupert: Radar plot using cumulative data to show absence of lounge movement Monday to Saturday. Absence is highlighted using a reverse scale such that no movement appears at the outer edge of the diagram and frequent movement appears at the centre.

**Figure 4:**
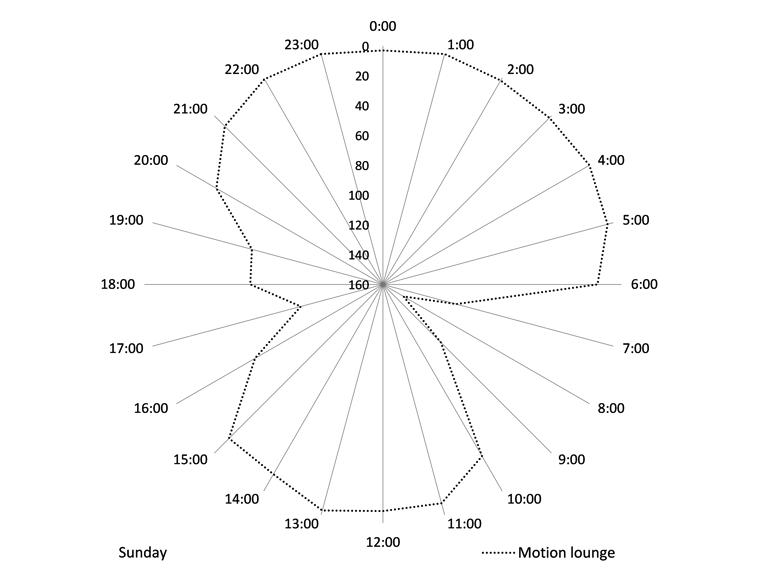
Rupert: Radar plot using cumulative data to show absence of lounge movement on Sundays. Absence is highlighted using a reverse scale such that no movement appears at the outer edge of the diagram and frequent movement appears at the centre.

Routine absence from home can be clearly identified on radar charts. Monday through Saturday, Rupert has lunch at the residential home restaurant. This regular absence from home is clear from Figures 3 and 4, which use a reverse scale to highlight absence of motion between 12 pm and 2 pm (Figure 3). On Sundays, however, he attends church and has lunch with his family. Figure 4 shows that he is away between 10 am and 3 pm. Understanding the individual’s cyclical absences from home help explain variance in data and can reduce the occurrence of false-positive alarms by distinguishing absence from home, from absence of movement due to a fall or other acute medical incident. Knowing an individual’s social routine also provides the opportunity to monitor routine activity outside the home using absence of movement.

#### Seasonal: Understanding the Impact of Weather and Daylight

Routines vary according to the seasons and weather. All residents spoke of changes to routines that were related to the temperature. For example, Elizabeth explained changes in hygiene practice:

(Shower time) can vary... in the winter time it will be before an evening meal... if you have your shower... just when the sun’s going on down, the room is still a bit warm, because (the sun is) on that side of the house... If you leave it until nine o’clock on a frosty night, it’s a lot colder in (the bathroom)... In the summer time I shower every day, but in the cold weather I get lazy and I think... I am not going out tomorrow I won’t shower tonight.

Elizabeth

In winter, Elizabeth also dresses later in the day. As she stays in her warm dressing gown longer, she also has hot porridge rather than muesli for breakfast. Temperature also affects her level of activity. The results show changes in the frequency of motion associated with temperature. Independent-samples *t* test showed significant differences in movement by room on the coldest and hottest days [33]. Frequency of motion sensor firings in the bedroom was significantly lower on cold days (Mean 34, SD 7.34) compared with hot days (Mean 41, SD 10.72), where *t*_92_*=* −3.6, *P=*.001) and similarly for the bathroom, cold (Mean 20, SD 12.88) compared with hot days (Mean 37, SD 25.39), where *t*_92_) *=* −4.19, *P=*.001. There was no significant difference for the kitchen or the lounge; however, her lounge room is fitted with reverse-cycle air-conditioning.

Above average high or low temperatures can lead to temporary changes in routine. Interview data indicated that Jacqui and Rupert changed their walking routine on hot days, either forgoing walks or changing the time of day. Hot weather can also affect the amount of movement in the home. Elizabeth is more likely to take an afternoon nap: “No, I’m not one to sleep very much, but over the hot weather... I have gone to sleep twice.”

Furthermore, the number of hours of daylight varies with seasons. Participants reported timing certain activities with sunset.

I mean, I – I walk regularly ... this time of the year I walk every evening just as the sun’s going down, for 20 minutes... I go for the walk according to the sun. At the moment I’m going about a quarter past seven ... and then (in winter) it will get to the point where I’ll go before dinner.

Elizabeth

Daylight savings also have to be accounted for in the data. In this study, because data showed clock time, motion and power use moved backward 1 hour as a result of the change. Clock shifts maintained scheduled activity at the same clock time, but caused an artificial shift in the time of sunrise and sunset, which must be allowed for in interpreting data and establishing alarms.

These cyclical annual changes affect routines and, consequently, the interpretation of sensor data. Factoring in an understanding of changes in routines that vary annually based on weather and seasonality will further help reduce false positives.

#### Trend Components: Detecting Gradual Change

Analysis of trend data exposed a gradual change. Using linear regression, and controlling for changes in temperature based on daily maximum temperature, the results showed a deterioration in Rupert’s sleep quality over the period of the trial. There was a significant increase in bedroom movement at night (beta *=*.059, *P*<.001) and a significant decrease in time between movements (beta *=* –3.692, *P*<.002). In parallel, Rupert’s activity during the day, as measured by the number of movements in the lounge, significantly decreased (beta *=* –.65, *P*<.003) with no significant change in the time between movements (beta *=* 1.029, *P*=.27). In other words, Rupert was increasingly restless at night, tossing and turning more frequently and more lethargic during the day. Adding a notification alert for changes to longitudinal data can be used to trigger preventive action.

#### Irregular Variation: Detecting Acute Events

After cyclical and seasonal changes have been accounted for and preference for routinization and adherence to routine taken into account, irregular variance in data is most likely the result of unexpected events, including illness, acute medical events, and falls. For ad hoc alerts to be effective in early detection and intervention, they need to be reliable. Analysis of the data showed the effect of variability of routines and looked at ways to establish customized thresholds to reduce false alerts.

To set up effective ad hoc alerts based on changes to routines, 2 conditions need to be met: activities should occur at the same time every day (consistency of timing), and routines should be a regular daily occurrence (adherence to routine). Examination of the data in this sample revealed that motion sensor data was more diffuse in time and therefore less reliable than power use data. Residents’ radar plots showed that kitchen power data and specifically breakfast power data were the most reliable, both in consistency of timing and in adherence to routine. Lunch and evening meals were less reliable indicators because residents reported regularly dining out as well as varying the time of evening meals according to what was on television. Knowing a person’s routine provides valuable insight into understanding variance in data, and together with radar plot, choosing appropriate triggers for alerts; for example, Jacqui revealed that:

On the weekend ... I’ll either have, bacon, eggs and ... during the week I usually have cereal, fruit, yoghurt, yeah and toast, always toast.

Jacqui

**Figure 5:**
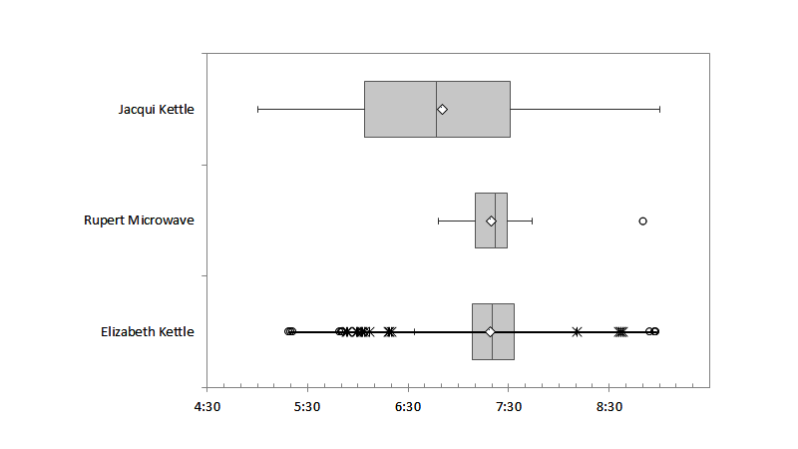
Breakfast preparation activity by participant. Appliance power use frequency of firing by time between 5:00 and 9:00. Only the appliance that is most consistently used for breakfast is illustrated. Boxes show first to third interquartile range (IQR), with the line of separation indicating the median and the diamond the mean. Whiskers indicate the minimum and maximum and outliers are represented by asterisk for values that fall between 1.5 and 3 IQRs and circles represent outliers that fall outside the 3 IQR.

Figure 5 summarizes the consistency of key kitchen appliance use at breakfast time. Jacqui is very consistent in her kettle use (no outliers), but she reports having several cups of tea in the morning, which accounts for the spread of data (IQR 1H27M, SD 1H01M). In contrast, Rupert is extremely precise about the time of microwave use (IQR 0H19M, SD 0H13M), whereas Elizabeth's coffee at breakfast, while quite fixed in time, has considerable variability, as indicated by the outliers, because she uses the kettle both before and after breakfast (IQR 0H25M, SD 0H26M).

Routines used to trigger ad hoc alerts should also be a regular daily occurrence. Adherence to routine was calculated based on the number of days when key appliance sensors fired within ±30 minutes of the median time of use (Figure 5). To triangulate the data, kitchen motion sensor firings (breakfast time midpoint ± 1 hour) are also represented. Details for all 3 participants are summarized in Table 3. Rupert ate breakfast between 7:30 am and 7:45 am. He was the most consistent in his habits with very high scores on adherence to routine for both morning microwave use (94.8%, 148/156) and motion in the kitchen (98.2%, 170/173). Elizabeth had breakfast between 7:15 am and 7:45 am. She also scored high on adherence to routine for kitchen motion at breakfast time, but she did not always use her kettle at the same time of day, scoring only 82.2% (120/145) on adherence to this routine. Jacqui generally ate breakfast between 7 am and 7:30 am. Her breakfast routine is the most variable of the 3 residents. She was frequently away from home (47 out of 181 days), and her adherence to routine for “morning kettle use” is low (63%, 62/98), but her “motion in the kitchen” score for adherence to routine is higher (85.7%, 102/119). The data showed both the variation in practice for different routines and the relative reliability of routines for use as alerts.

**Table 3:** Adherence to breakfast routines.

Description	Jacqui		Rupert		Elizabeth	
	Kettle	Kitchen	Microwave	Kitchen	Kettle	Kitchen
Days absent	47^b^	45^b^	1	1	11	10
Days with data^a^	98	119	156	173	145	145
Days breakfast not taken	36	17	8	3	25	8
Total days with data	181	181	165^c^	177^c^	181	166
Adherence to schedule^d^, n (%)	62 (63.2)	102 (85.7)	148 (94.8)	170 (98.3)	120 (82.8)	137 (94.5)

^a^± 0.5 hours of time of the median calculated on all power use between 5 am and 9 am .^b^50 days absent, but on 4 of the days, breakfast was prepared on day of departure.^c^Data missing because sensor not installed till after start date.

^d^Daily adherence to schedule based on median ± half an hour.

The case studies also show 2 examples of ad hoc events that can be clearly traced by changes to routine, illness, and death of spouse. Someone who is ill may stay in bed, stay seated for longer, and forgo regular meals. These changes are detectable with motion and power sensors. Additionally, self-reported information from residents or relatives can narrow down how residents respond to being unwell. Rupert’s daughter-in-law, who has remote access to his daily movement data via a secure website, was able to identify when he was ill because of the reduced movement in the kitchen:

I look at the kitchen and I know when he’s sick because it’s down, the usage (motion data) ... And I know definitely when he’s sick he doesn’t cook for himself.

Rupert’s daughter-in-law

**Figure 6:**
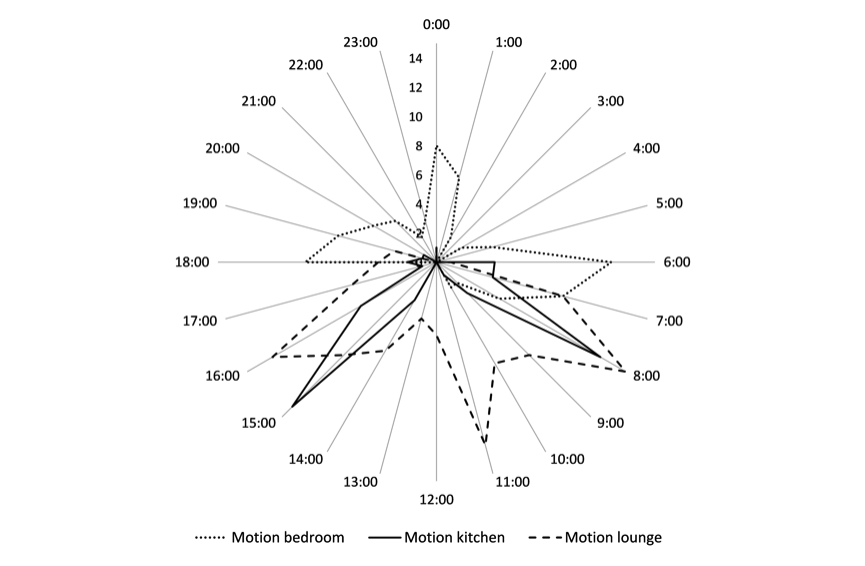
Rupert: Normal household movement Tue & Wed Week 3.

**Figure 7:**
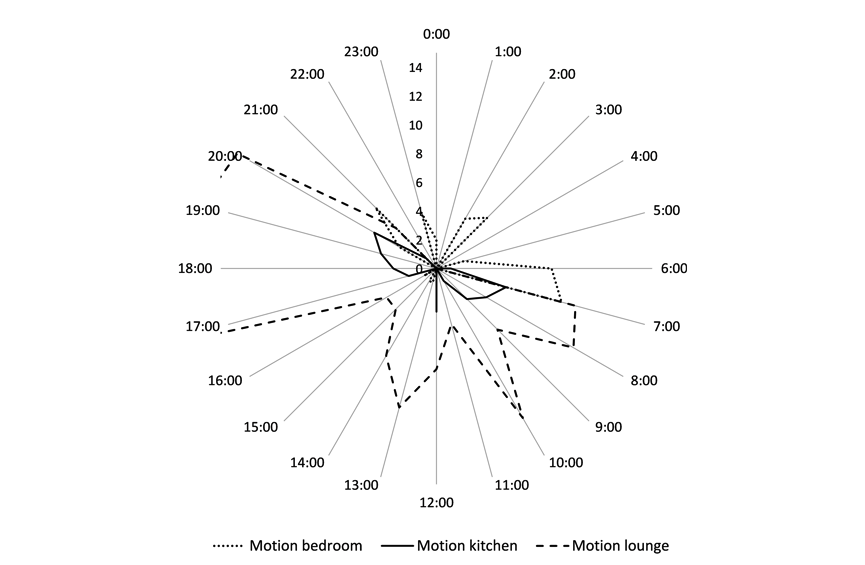
Rupert: Changes in motion due to (assumed) illness. Unusual houshold movement attributed to illness Tues & Wed Week 4.

The first example is an example of illness. On Wednesday and Thursday of Week 3, it appeared that Rupert was unwell. Rupert’s normal routine for the same 2 days of the preceding week is shown in Figure 6. Week 3 (Figure 7) is considerably different. Rupert did not go up to the restaurant for lunch as normal, and on the Thursday, he did not use the microwave in the morning for breakfast. There were also different patterns of movement around the home on those 2 days. Movement in the kitchen and bedroom was considerably less than normal. Movement in the lounge was different in both the amount and the timing of movement. Movement in the lounge was considerably higher, the peak of movement in the lounge was later, unusually high, and more prolonged than usual. A possible explanation for this high level of movement in the lounge at that time is that perhaps his family and possibly a doctor had come to visit. A visual inspection of the routine data showed that although his routine had changed he was still moving about the house and spending most of his time in the lounge, which suggested that he was unwell but coping.

**Figure 8:**
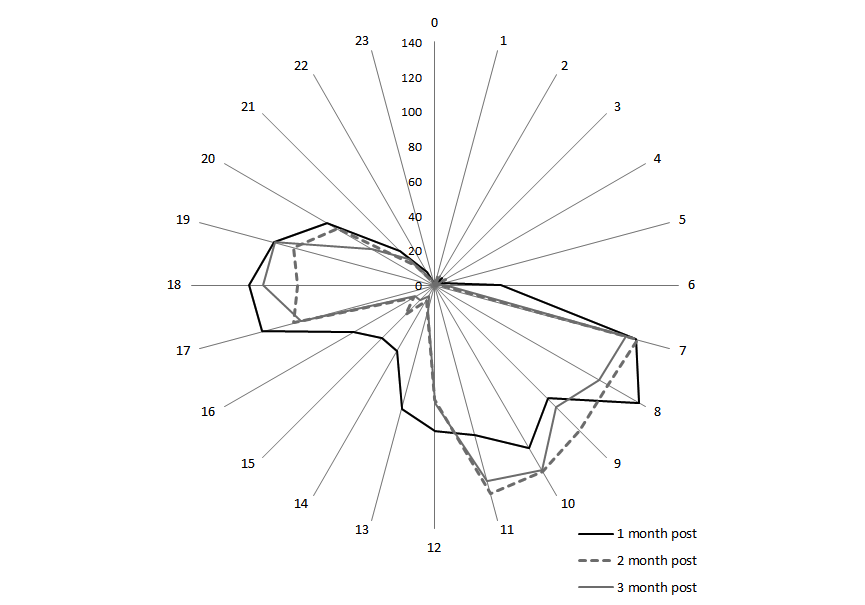
Elizabeth: Changes in lounge movements on bridge club days (Mondays, Thursdays, and Saturdays) 1, 2, and 3 months after the death of her husband showing a return to routine (resumption of bridge club) around Month 2.

The second example is a more persistent change in daily routine. Elizabeth lost her spouse around the time the sensors were first installed. Traumatic events can result in a temporary loss of routine [34]. Therefore, monitoring return to normal can provide evidence of recovery from trauma. The data suggested that Elizabeth did not regularly attend bridge club (1 pm to 5 pm) in the first month following the death of her husband, but resumed her regular attendance around Month 2 (Figure 8). This indicated that with foreknowledge of routines, sensor data could be used to unobtrusively monitor changes to socialization through changes to normal routines.

The main challenges for using sensor data as ad hoc alerts are individual’s preference for routinization, adherence to routine, and consistency in timing. Understanding these variations and the cyclical and seasonal variation in timing of routines is important in determining the thresholds for alerts. Having a two-sensor alert (power and motion) could be additional security against false positives and technical issues. Additionally, it is possible to calculate the longest time spent in the room such as the bathroom and the kitchen, rooms, that have very specific purpose, and to set up an alert when maximum length of stay is exceeded as another opportunity for cross-checking alerts related to change in routines.

## Discussion

### Principal Findings

This study demonstrated how a routine-based approach could help make sense of the large amounts of data from in-home sensors in such a way as to make the interpretation of data easy, intuitive, and transparent. The routine-based approach can be used to detect both change in sensor data and absence of sensor data for routines that take place outside the home, facilitating the use of alerts for both ad hoc events and gradual change over time. Importantly this study advances understanding of how sensor data can be used in real-world applications for real-time monitoring through an improved understanding of normal variation in routines and behavior (cyclical, seasonal) and a better understanding of the need for customized setups to allow for inter- and intraindividual differences in preference for routinization and in timing of and adherence to different routines. Overall these results support the use of in-home sensors as an extension of existing health care prevention approaches, one which, because of the 24/7 nature of the data, has the potential to facilitate aging in place.

This study builds on earlier work by Kaye et al [11] and others [35] and extends prior research by demonstrating through case studies, specific instances of how routines can be used to monitor the health and well-being of the elderly, and to identify factors that need to be controlled for to improve reliability of interpretation of sensor data for real-world applications.

Routines are an appropriate framework for understanding sensor data because they account for the cyclical nature of everyday living and because changes to routine, including the timing, are important in understanding both physical and psychological well-being [16,36-38]. Routines are particularly appropriate for monitoring the elderly because preference for routinization increases with aging [36,39]. The presentation of data in a 24-hour clock format is important because it includes timing of activities. It is not sufficient to have the skills or the ability to undertake the activities of daily living, but it is also necessary that they be organized into regular routines [14,15]. The 24-hour clock format also makes understanding and interpreting each day’s activity logical and intuitive.

This research also reflected on the challenges of interpreting sensor data for the development of practical applications based on single-point comparisons. Viewing data as cyclical increases the reliability of single-point comparisons by facilitating comparisons of like for like, that is, comparing routines on any given day with the matching activities of the same day in previous weeks. Challenges for interpreting data include allowing for predictable and unpredictable events arising from the local context. First, cultural routines and seasonal change are contextual factors that shape activity over time. Allowance needs to be made for cultural routines such as weekends, holidays, religious practice, as well as seasonal changes of temperature and daylight [40,41]. Second, as noted by Ohta et al [6], the elderly are sensitive to changes in temperature [42]. By incorporating weather data into the system, it is possible to account for the unpredictable effects of precipitation and temperature on daily activity. Knowing, through interview, how temperature and precipitation affect a person's daily routines can further reduce false-positive alerts.

Importantly, prior knowledge of routines and variations in routine personalizes information that is otherwise just numbers. This is especially important if the sensor data are to be used by community services providers who can better know the individuals they care for through the everyday occupations and routines that give structure and meaning to life [31]. Knowledge of routines can be gathered by completion of a Web-based form at the time of installation of sensors, updated as needed, and used to annotate graphical presentation of data in the 24-hour polar charts. Furthermore, prior knowledge of routines makes it possible to rapidly establish ground truth of sensor data and accelerate the establishment of a personalized baseline against which changes in routine can be measured. Finally, only prior knowledge of routines can provide an understanding of activities outside the home and how they contribute to the well-being and quality of life, which could then be used to evaluate how time is apportioned to measure successful aging [43].

Future research should look at testing algorithms to monitor real-time daily activity using data from the same day of the week to allow for normal weekly variation in routine and, based on a 4- to 6-week moving window, to allow for changes in seasonal movement and activities. Daily weather data would need to be incorporated, such as profiles to allow for unseasonal above or below average temperatures as well as precipitation, to account for normal weather-based changes to routine. Data from different sensors should be correlated, for example, kitchen appliance use and motion in kitchen to detect battery failure or technical faults and minimize false alerts. Incorporating room layout and location of exits can additionally allow for interpretation of movement around the home. A two-level alert system, low and high, based on deviation from normal routine could be established. Alerts should be accompanied by a summary of the issues: routine versus actual and a time line of activities. Changes in behavior over time for comparable seasons can look for changing trends.

**Figure 9:**
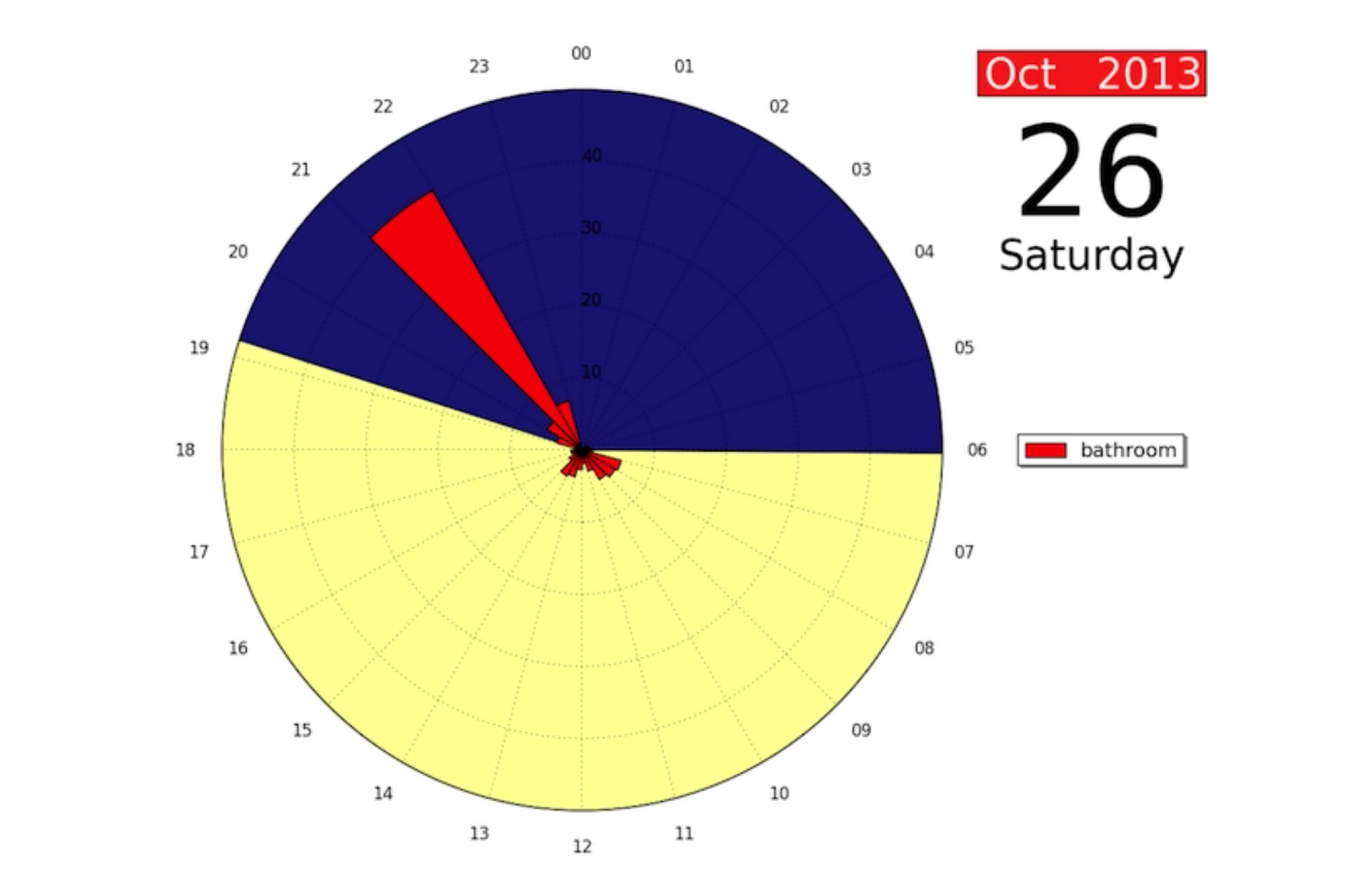
Screenshot of user interface showing daily bathroom movement over a period of 4 months using a 24-hour clock format.

The user interface should display the 24-hour clock showing movement for key rooms (bedroom, kitchen, bathroom, lounge). Figure 9 shows an example of a user interface showing 24 hours of bathroom movement. An animated version showing 4 months of data can be found in Multimedia Appendix 1. For individuals whose routines differ from day to day, daily data need to be juxtaposed with comparable days of week in order to identify changes in routine. Users of the system should be allowed to adjust alert sensitivity to allow for variation in preference for routinization as well as variation between room use.

### Implications

In-home sensors may be able to facilitate aging in place and improve community health care services through the provision of alerts and notifications for early intervention as well as longitudinal health data for improved decision making. They could also potentially complement and reduce the direct costs of care in the community by facilitating routine monitoring. For smart homes to be a reality, it is important to strike a balance between the privacy and independence of the elderly and the effectiveness of monitoring systems. Furthermore, it is important that in-home monitoring provides data that allow carers and relatives to engage with and understand how these technological solutions can enhance care for the elderly, and that these systems are not viewed as a substitute for face-to-face health care, but as a means of improving the effectiveness of current interventions.

### Limitations

Although research based on case studies is not generalizable, the results nonetheless provide rich insight into how routines can be used to monitor data in smart homes. There also are limits to the interpretation of data, especially from motion sensors. Most problematic is dual occupancy because sensors cannot distinguish between individuals, and neither can they separate out the activity of visitors to the home. However, new motion sensors under development may overcome this problem. Sensors cannot distinguish between types of motion. Therefore, matching specific daily activities such as getting dressed or doing daily exercises can only be inferred by time of day and room use. Equally because lack of motion could be due to absence from home or a fall, accurate and early fall detection is problematic for motion sensors [5,6,11]. However, new more sophisticated sensors can improve motion detection [44], and some, if not all, of these challenges can be overcome with the inclusion of additional sensors in the home, such as reed switches on doors and cupboards, GPS trackers on key rings, and through data mining or machine learning techniques from data science.

Concerns about ethics with respect to informed consent for technology-based research in the elderly remain problematic. There were some erroneous perceptions by residents who did not fully understand the technology, despite repeated explanations. This was mitigated by involving the relatives of residents in the process. However, it is imperative for smart homes pilots and installations for the elderly to continue to clearly communicate what the technology can do and what it cannot do, to better allay concerns and manage expectations.

### Conclusions

This study demonstrated the usefulness of a routine-based approach for making sense of smart home data and furthered the understanding of the challenges that need to be addressed in order to make real-time monitoring a reality, through improved visualization of data and a better understanding of variation in routine, which could improve the effectiveness of alerts and notifications. Future research needs to explore larger longitudinal datasets to test the potential of routines for alerts in order to minimize false positives and negatives and still be able to deliver reliable alerts to the satisfaction of persons being monitored and their carers.

## Appendix

Multimedia Appendix 1 Animation showing 4 months of daily bathroom movement data on a 24-hour clock format.

## Abbreviations

AEDT: Australian Eastern Daylight Time
AEST: Australian Eastern Standard Time
GMT: Greenwich Mean Time
KwH: Kilowatt hour
